# *Rhizobium laguerreae* Improves Productivity and Phenolic Compound Content of Lettuce (*Lactuca sativa* L.) under Saline Stress Conditions

**DOI:** 10.3390/foods9091166

**Published:** 2020-08-24

**Authors:** Miguel Ayuso-Calles, Ignacio García-Estévez, Alejandro Jiménez-Gómez, José D. Flores-Félix, M. Teresa Escribano-Bailón, Raúl Rivas

**Affiliations:** 1 Departamento de Microbiología y Genética, Universidad de Salamanca, Edificio Departamental de Biología, 37007 Salamanca, Spain; miguelac96@usal.es (M.A.-C.); jdflores@usal.es (J.D.F.-F.); raulrg@usal.es (R.R.); 2 Spanish-Portuguese Institute for Agricultural Research (CIALE), 37185 Salamanca, Spain; 3 Grupo de Investigación en Polifenoles (GIP), Departamento de Química Analítica, Nutrición y Bromatología, Facultad de Farmacia, Universidad de Salamanca, 37007 Salamanca, Spain; igarest@usal.es (I.G.-E.); escriban@usal.es (M.T.E.-B.); 4 Associated Unit University of Salamanca CSIC (IRNASA), 37008 Salamanca, Spain

**Keywords:** bioactive compounds, phenolics acids, flavonols, PGPR, lettuce, food quality

## Abstract

Lettuce (*Lactuca sativa* L.) is a widely consumed horticultural species. Its significance lies in a high polyphenolic compound content, including phenolic acids and flavonols. In this work, we have probed the ability of *Rhizobium laguerreae* HUTR05 to promote lettuce growth, under in vitro and greenhouse conditions (both non-saline and saline conditions). This strain has shown several in vitro plant growth promotion mechanisms, as well as capacity to colonize lettuce seedlings roots. We have analyzed the effect of the rhizobacterium inoculation on mineral and bioactive compounds in lettuce, under greenhouse conditions, and found a rise in the content of certain phenolic acids and flavonoids, such as derivatives of caffeoyl acid and quercetin. The genome analysis of the strain has shown the presence of genes related to plant growth-promoting rhizobacteria (PGPR) mechanisms, defense from saline stress, and phenolic compound metabolism (such as naringenin-chalcone synthase or phenylalanine aminotransferase).

## 1. Introduction

The current trend of consumers to maintain a healthy and balanced diet has led to an increase in the consumption of fresh vegetables in recent years, due to their low caloric content and richness in bioactive compounds, which are the secondary metabolites with remarkable effects on human health [1,2,3].

Lettuce (*Lactuca sativa* L.) is a species belonging to the Asteraceae family, widely cultivated in temperate areas around the globe, more than 21 million tons are produced annually [4]. It is traditionally consumed fresh, as in a Mediterranean diet. Lettuce stands out for its content of fiber, folate, vitamins, and bioactive compounds such as phenolic acids and flavonoids, mainly flavonols [1,2,5]. 

Phenolic acids, an important subgroup within phenolic compounds, are characterized by possessing a carboxylic acid group with two distinctive structures: the hydroxybenzoic and hydroxycinnamic structures [6]. On the other hand, flavonols are a flavonoid subclass of a different group of phenolics compounds, whose basic chemical structure consists of C6–C3–C6 rings with different substitution patterns [6]. Several studies indicate that these polyphenolic compounds have beneficial effects on human health, conferring protection against cardiovascular diseases and various types of cancer. These benefits are classically associated not only with their antioxidant activity but also with their anti-inflammatory and, even, antigenotoxic properties [1,3,7,8]. 

Nowadays, the yield of horticultural crops is being severely affected by adverse environmental conditions (such as soil salinity), which are associated with the impact of climate change that in turn is caused by the current model of energy production and consumption [9,10]. Soil salinity is considered one of the main causes of abiotic stress in plants due to the various negative physiological responses that are generated: osmotic stress, nutrient deficiency, toxicity, and lower resistance to pathogens [11,12,13]. In addition, the salinization rate increases annually by 10%, and it is expected that 50% of arable land will contain high salt concentrations by 2050 [14]. According to the literature, a soil is considered saline when it has an electrical conductivity of the saturation extract higher than 4 dS/m (40 mM NaCl, approximately) [15]. Many horticultural crops stand out for their sensitivity to salinity, including lettuce, whose tolerance threshold for this type of abiotic stress is 1.3 dS/m [16].

Therefore, the adverse environmental conditions, together with the increase in the world population and its associated demand for food consumption, generate a complex scenario for the development of agriculture, in which it is absolutely necessary to find new methodologies to increase the crops, even under stress conditions, in an environmentally friendly manner. One of the alternatives that poses more potential to ensure productivity and sustainability within the field of agriculture is based on the use of plant growth promoting rhizobacteria (PGPR) capable of being used as biofertilizers, which, in turn, are naturally found in the soil. These bacteria favor crop yields by increasing the productivity and nutritional content of the plants with which they are associated, even in adverse environmental conditions [17,18,19,20].

It is important to note and consider that biofertilizers must be safe products for human health and the environment. Among the innocuous microorganisms with PGPR potential, the group of rhizobia stands out not only due to its ability to fix atmospheric nitrogen in symbiosis with legumes but also due to its capacity to promote plant development and enhance food quality in non-legumes [17,18]. In this sense, the bacterial genomic analysis constitutes a fundamental and useful tool to develop a conceptual framework on plant–microorganism interaction and its beneficial effects [21]. 

Thus, the objective of this study was to determine, for the first time, the ability of a rhizobia strain to act as a promoter of lettuce growth and to improve lettuce content in bioactive compounds.

## 2. Materials and Methods

### 2.1. Bacterial Strain and Green Fluorescent Protein (GFP) Labeling

The HUTR05 strain used in this work was isolated from the inside of surface-sterilized effective nodules of *Trifolium repens* L. grown in a soil in the province of Salamanca (Spain): Huerta (40°58′13.0″ N 5°28′18.0″ W), using the standard method of Vincent (1970) on YMA (yeast mannitol agar) plates at 28 °C. 

A green fluorescent protein (GFP)-tagged strain was obtained by biparental mating using *Escherichia coli* S17.1 as a donor strain, which carried plasmid pHC60, as described in García-Fraile et al. [22]. The recombinant strain was routinely grown at 28 °C in TY (tryptone yeast extract) medium and *E. coli* S17.1 in Luria–Bertani (LB) at 37 °C, both mediums supplemented with tetracycline (10 µg mL^−1^).

### 2.2. Phylogenetic Analysis

The classification of the HUTR05 strain at genus level was performed through the amplification and sequencing of 16S rRNA genes following the protocol described by Rivas et al. [23]. Phylogenetic classification at species level was carried out through the concatenation of the *atpD* and *recA* genes, whose amplification and sequencing were performed according to Flores-Félix et al. [24]. The obtained sequences were compared with those from GenBank using the BLASTN program [24].

### 2.3. HUTR05 Genome Sequencing and Annotation

ZR (Zymo Research) Fungal/Bacterial DNA MiniPrep was used to obtain the genomic DNA for genome sequencing from pure bacterial cells of HUTR05 strain, which were grown on TY plates and collected after 24 h at 28 °C. Draft genome sequence of the isolated cells was obtained by shotgun sequencing on an Illumina MiSeq platform via a paired-end run (2 × 251 bp). The genome sequence data were assembled using Velvet 1.2.10 [25]. Gene annotation was performed using RAST 2.0, and SEED-viewer 2.0 framework was used to search for genes [26].

### 2.4. Analysis of In Vitro PGPR Mechanisms

Two different forms of the Pikovskaya agar medium [27] were used to evaluate the ability to solubilize insoluble forms of phosphate (P), each of which had a different P source (bicalcium and tricalcium). The National Botanical Research Institute’s Phosphate (NBRIP) growth medium (with hydroxyapatite as the phosphate source) [28] was also used to evaluate this ability. The experiment was performed three times.

Siderophore production was analyzed using M9-CAS-AGAR medium [29]. The strains were inoculated in this medium for five days at 28 °C and, then, examined for the presence of yellow–orange halos around the colonies. 

Indole acetic acid (IAA) production was measured in JMM medium (John Howieson minimal medium), supplemented with tryptophan (0.17 g L^−1^) according to the protocol described by García-Fraile et al. [22]. Additionally, IAA production was also detected by HPLC, as described in Jimenez-Gómez et al. [18].

### 2.5. Colonization Assays and In Vitro Effect on Plant Growth

Lettuce (*L. sativa* L.) var romaine seeds were surface-sterilized by immersion in ethanol 70% for 30 s and sodium hypochlorite solution (5%) for 5 min [30], followed by 5 washes with sterile distilled water and germinated on water-agar plates, keeping them in darkness for 24 h. Subsequently, the seedlings were transferred to square water-agar plates (12 × 12 cm), placing 5 seedlings per plate. Two different experiments were prepared with 15 seedlings for each type of inoculation: the first experiment was performed by inoculating the unlabeled strain and the second experiment, inoculating with this HUTR05 GFP-tagged strain, in order to determine the effect on early plant growth and the colonization assays, respectively. Each seedling was inoculated with 150 µL of a bacterial suspension with an Optical Density (600 nm) of 0.6 (10^8^ CFU mL^−1^). To prepare the bacterial suspension, the strain was grown at 28 °C for 48 h on YMA plates. In addition, for each experiment, a negative control was arranged with the same number of seedlings, inoculated with 150 µL of sterile distilled water. For early plant growth assays, the seedlings were maintained in a growth chamber and observed at seven and fourteen days post-inoculation (dpi); at these times, the length of the shoots and the roots and the number of leaves and secondary roots of seedlings inoculated with unlabeled strain were measured. The experiment was performed at least three times. Therefore, 90 plants per treatment and analysis day were collected for obtaining the data. 

Moreover, colonization assays were observed seven days post-inoculation using a NIKON eclipse 80i fluorescence microscope with a mercury lamp associated (Nikon, Tokio, Japan). For microscopic analysis, the roots of the seedlings inoculated with GFP-tagged strain were washed with sterile distilled water and stained with a solution of propidium iodide (10 µM). The experiment was performed three times.

### 2.6. Growth Promotion and Saline Tolerance Assays in Plants

The ability of strain HUTR05 to promote the growth of *L. sativa* L. var romaine and to alleviate saline stress was evaluated by using a mix of non-sterilized soil and vermiculite “SEED PRO 6040”/vermiculite (3:1 *v*/*v*) (PROJAR, Madrid, Spain) as substrate, under greenhouse conditions. The surface-sterile seeds were germinated on water-agar plates and transferred to plastics pots containing 4.8 L of substrate, subsequently. After seven days, each seedling was inoculated with 5 mL of the strain suspension with a final concentration of 10^8^ CFU mL^−1^. The suspension was prepared by growing the strain on YMA plates for 48 h at 28 °C and then suspending it in sterile water. Fifteen plants were included in each treatment: an inoculated control and inoculation with HUTR05. Seedlings were divided into two assays, one of them developed under normal conditions, and the other one under saline stress conditions, in order to determinate the ability to promote plant growth and to relieve such stress, respectively. To develop saline stress conditions, plants were irrigated with an aqueous solution of NaCl (100 mM) every 48 h, whereas the plants developed under normal conditions were irrigated only with water, under the same conditions. Plants were maintained in a greenhouse for 27 days, (50–60% relative humidity, temperature between 20 and 25 °C, photoperiod 16/8). After that time, several parameters related to the aerial part of this horticultural species were analyzed, considering it is the marketable part of the crop and is used for consumption: number of leaves, wet and dry weight of the aerial part, and chlorophyll content of the leaves with a chlorophyll meter SPAD-502PLUS (Soil Plant Analysis Development) (Konica Minolta, Osaka, Japan). The experiment was performed three times. Therefore, 180 plants per treatment and growth condition were collected for obtaining the data.

Plant samples were analyzed in the Analysis and Instrumentation Service at IRNASA-CSIC (Salamanca, Spain). Total carbon and nitrogen were both determined by the Dumas method using a LECO CN628 Combustion Analyzer. Mineral composition in plant tissues was determined by inductively coupled plasma optical emission spectrometry (ICP-OES, Varian 720-ES, Mulgrave, Australia) after digestion in a microwave oven (Milestone ETHOS UP, Sorisole, Italy) using diluted nitric acid and hydrogen peroxide.

### 2.7. Phenolic Analysis of Lactuca sativa L. Leaves

The phenolic composition was determined as previously reported [20]. Briefly, plant samples were freeze-dried and, then, 5 mg were extracted using MeOH:H_2_O 80:20 (8 mL) in a bath of ultrasounds (30 min, three times). The supernatants were combined, and chlorophylls were removed by liquid–liquid extraction using hexane. The final extract was concentrated under reduced pressure to reach a final volume of 2 mL.

Analyses by high performance liquid chromatography coupled to diode array detection and mass spectrometry (HPLC-DAD-MS) were performed to determine the phenolic composition of plant extracts. Samples were injected in a Hewlett-Packard 1200 series liquid chromatograph (Agilent Technologies, Waldbronn, Germany) using a Spherisorb^®^ S3 ODS-2 C18 reversed phase, 3 µm, 150 × 4.6 mm column (Waters Corporation, Milford, MA, USA) thermostatted at 35 °C. An aqueous solution of formic acid (1 mL L^−1^) (A) and acetonitrile (B) were used as solvents following the gradient previously reported [20]. Detection was carried out at 280, 330, and 370 nm as preferred wavelengths and spectra were recorded from 220 to 600 nm. The mass spectrometer was connected to the HPLC system via the DAD cell outlet. MS detection was performed using an API 3200 Qtrap (Applied Biosystems, Darmstadt, Germany) equipped with an electrospray ionization (ESI) source and a triple quadrupole-ion trap mass analyzer and employing the previously developed methodology for non-anthocyanin compounds [8]. Briefly, zero grade air was used as the nebulizer gas (30 psi) and turbo gas (40 psi), 400 °C and nitrogen served as the curtain (20 psi) and collision gas (medium). The ion spray voltage was set at −4500 V in the negative mode and spectra were recorded in negative ion mode between m/z 100 and 1700. The MS detector was programmed to perform an enhanced MS to show full-scan spectra followed by an enhanced product ion (EPI) to show the fragmentation pattern of the main detected compound. 

Compound identification was performed based on the UV–vis spectra, retention times, and mass spectra, by comparison with the commercial standards when available. Individual compounds were quantified from the peak area values obtained in the chromatograms recorded at 280 nm (protocatechuic acid glucoside), 330 nm (caffeic acid derivatives), or 360 nm (flavone and flavonol derivatives) using the external standard method: coumaric acid, quercetin 3-*O*-glucoside, and luteolin 7-*O*-glucoside were quantified by using a calibration curve of their own commercial standards. The contents of caffeic acid derivatives, protocatechuic acid glucoside, apigenin derivative, and quercetin derivatives were expressed as caffeic acid, protocatechuic acid, apigenin 7-*O*-glucoside, and quercetin 3-*O*-glucoside equivalents, respectively. The results, expressed in g kg^−1^ of plant dry weight, were the mean value of three independent analyses.

### 2.8. Statistics

Data were analyzed using StatView 5.0 software (BrainPower Inc, Fremont, CA, USA), carrying out the one-way analysis of variance. Mean values were compared with Fisher’s protected least significant differences (LSD) test (*p* ≤ 0.05). Two-way analysis of variance (ANOVA) was performed to analyze the effect of salinity and inoculation on the total content of phenolic acids and flavonoids by using GraphPad Prism 8.0 software (GraphPad Software, San Diego, CA, USA).

## 3. Results and Discussion

### 3.1. Isolation and Phylogenetic Classification of HUTR05 Strain

The 16S rRNA gene sequence comparison showed that the HUTR05 strain had a similarity of 99.8% with respect to *Rhizobium laguerreae* FB206^T^. Moreover, the two housekeeping genes, *recA* and *atpD*, were also analyzed in the phylogenetic relationships in *Rhizobium* genus [24], in this case, the analysis of the concatenated gene sequences showed that HUTR05 had a 100% similarity with *R. laguerreae* FB206^T^. The species *R. laguerreae* has also been described by developing nodules in other legumes, such as *Vicia faba* and *Phaseolus vulgaris*, as well as by the potential as a growth promoter in interesting horticultural crops [18,31]. However, this is the first time that the effects of the inoculation of this bacterial species on the plant promotion and nutritional quality of lettuce have been evaluated.

### 3.2. Analysis of In Vitro PGPR Mechanisms

The strain HUTR05 showed phosphate solubilization halos around a bacterial colony of about 2 mm radius, in Pikovskaya agar medium with Ca_3_(PO_4_)_2_, this ability was not demonstrated when CaHPO_4_ or Ca_5_(PO_4_)_3_OH were used as P source. This strain also developed a 7.5 mm yellow–orange halo around the bacterial colony in M9-CAS-AGAR medium, indicating its capacity of siderophore production. In addition, HUTR05 produced 71.0 mg·L^−1^ of indole acetic acid, when grown in JMM liquid medium supplemented with tryptophan; whereas when measuring the concentration of this phytohormone by HPLC, the result obtained were 1.34 mg·L^−1^.

These results indicate that HUTR05 strain, established within the *Rhizobium laguerreae* species, has various PGPR mechanisms in vitro: secondary metabolite production (IAA and siderophores) and nutrient mobilization ability (phosphorus). These results are consistent with alternative results obtained in various studies conducted with other strains within this same genus [18,22]. Based on the IAA results obtained by HPLC, it is worth highlighting the ability of the HUTR05 strain to produce that phytohormone, presenting higher concentrations than other strains belonging to the same species and whose analyses were performed under the same conditions [19]. These results allow us to determine that the strain HUTR05 could have a high potential in plant growth promotion, although the subsequent verification in plant assays is also required.

### 3.3. HUTR05 Draft Genome Analysis

The development of genomic sequencing and mining has revolutionized the field of microbiology becoming a fundamental tool for the analysis and improvement of knowledge related to microbiological processes, such as microorganism–plant interactions [21]. Genome analysis allows the identification of genetic characteristics, as well as the relationships among its properties as bacterial biofertilizers [20]. The HUTR05 draft genome is composed by 156 contigs with a genome length of ~7 Mbp, 7374 predicted coding sequences, and G + C content of 60.7%. The genome sequence of *R. laguerreae* HUTR05 was deposited in DDBJ/EMBL/GenBank under the Bioproject PRJNA603738 (accession number JABEQX000000000).

Based on in silico analysis, the HUTR05 genome showed genes involved in uptake and transport of necessary nutrients not only for bacterial but also for plant growth development. The HUTR05 genome contains the PHO operon, formed by genes related to inorganic phosphate binding (*PstS*) and membrane transport (*PstACB*), as well as enzymes related to the solubilization of insoluble forms of phosphate, such as phosphatases (EC 3.6.1.11, EC 3.6.1.10, and EC 3.1.3.1) or citrate synthase (EC 2.3.3.1). The HUTR05 genome also encoded genes involved in siderophore biosynthesis, such as aerobactin (*iucA* and *iucC*) or anthrachelin (*siderX1*, *siderX2*, *siderX3*, *siderX4*, *siderX5*, and *siderX6*), an efficient mechanism for chelation of iron and the increase of the bioavailability of this nutrient for plants and microorganisms production, inhibiting the growth of other competing bacteria or fungi [19]. Moreover, the presence of enzymes related to different biosynthesis pathways of indol-3-acetic acid in the genome of this strain was observed, indole-3-glycerol phosphate synthase (EC 4.1.1.48) and aliphatic amidase (EC 3.5.1.4). In this sense, bacterial phytohormone production is consider an important phytostimulation mechanism due to the effects it has on plant development and growth enhancement under stressed conditions [9]. Furthermore, the HUTR05 genome encodes genes involved in exopolysaccharide (*exoF*, *exoQ*, and *exoZ*) and lipopolysaccharides (*wadC*, *lptABC*, and *lptG*) biosynthesis, as well as the enzyme coding genes related to cellulase production (EC 3.2.1.4), both polymers required for biofilm formation [32,33].

Soil salinity causes negative effects in rhizobacteria due to the osmotic stress and the toxicity of ions. However, salt-tolerant strains are able to survive and colonize the rhizosphere under salt stress conditions [13]. The main mechanisms of salt tolerance in bacteria are based on the cytoplasmic accumulation of osmolytes, such as proline, glycine-betaine, and trehalose [13,14]. In the genome of HUTR05 the presence of genes related to transport and biosynthesis of these solutes, *OpuAABC* and *ProVWXZ* transport systems, *betB* (betaine aldehyde dehydrogenase (EC 1.2.1.8)), and treS (trehalose synthase (EC 5.4.99.16)), was observed.

It has been widely studied that the biosynthetic pathway of phenolic compounds begins with the amino acid phenylalanine, which serves as a key point for the synthesis of phenolic acids and flavonols [6,7]. In this sense, the HUTR05 genome encoded genes involved in phenylalanine biosynthesis, such as prephenate dehydratase (EC 4.2.1.51), phenylalanine aminotransferase (EC 2.6.1.9), or aromatic amino acid transaminase (EC 2.6.1.57). The presence of the enzyme EC 2.3.1.74, a naringenin-chalcone synthase, was also observed in the HUTR05 genome, which is involved in obtaining naringenin from phenylalanine, a flavone that acts as a precursor in the synthesis pathways of kaempferol, quercetin, and apigenin [6]. According to the genomic analysis of HUTR05, it would be possible that the bacteria could act in a synergic way to increase the synthesis of plant phenolic compounds, although it has previously been suggested that rhizobacteria through the elicitation of the systemic tolerance process induced (IST) may induce changes in the content of plant phenolic compounds, even under saline stress conditions [9].

Therefore, these results allow us to determine the potential of this strain as a plant growth promoter, posing as a susceptible candidate for its use as biofertilizer, due to the presence of important genes related to plant–microorganism interaction, plant development, and food quality.

### 3.4. Roots Colonization Assays and In Vitro Growth Promotion of Lettuce

**Figure 1:**
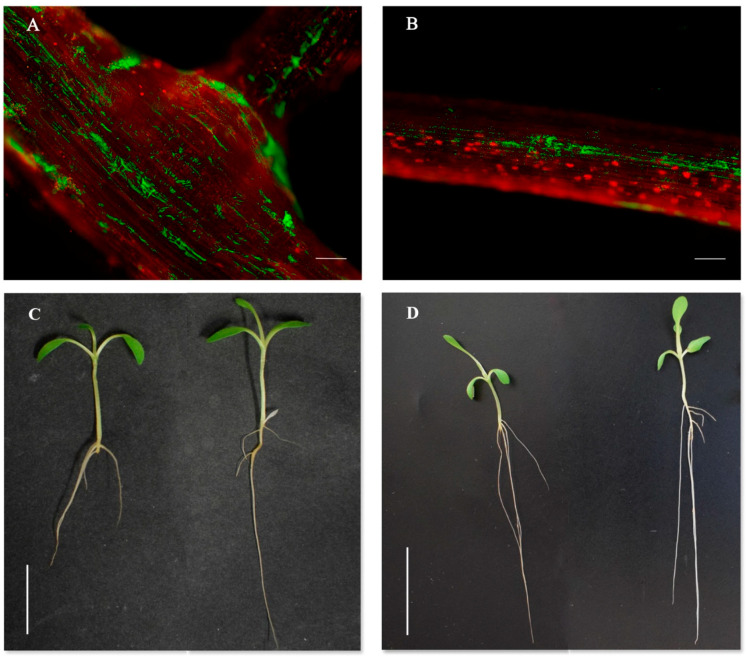
At the top, fluorescence optical micrographs of lettuce seedlings roots inoculated with HUTR05 green fluorescent protein (GFP)-tagged strains contrast stained with propidium iodide, seven days post inoculation: (**A**) show the ability of HUTR05 to colonize lateral root primordia (bar 250 µm); (**B**) show the biofilms formation of HUTR05 (bar 320 µm). At the bottom, general appearance of the seedlings (**C**) 7 dpi and (**D**) 14 dpi. Uninoculated control on the left and treated with HUTR05 on the right. Scale bars (**C**) 15 mm and (**D**) 30 mm.

Seven days post-inoculation, the GFP-tagged HUTR05 strain showed homogeneous colonization throughout the entire root and even showed growth in areas close to lateral root primordia (Figure 1A). Moreover, the selected strain was able to develop biofilms associated with epidermal intercellular spaces (Figure 1B). These polysaccharide structures have been described by their important role in salinity stress bacterial tolerance [9], so HUTR05 could have positive effects on the growth of lettuce plants, even in saline conditions. 

A more agile development during the early stages of plant growth is a fundamental factor to improve crop productivity. Lettuces inoculated with HUTR05 strain showed increases in the number of leaves and root and shoot length after seven days of inoculation (10%, 17%, and 7%, respectively) (Figure 1C), fourteen days post-inoculation the number of leaves was also 6% higher than that in the control, as well as the number of secondary roots, 24% statistically significantly higher (Table 1) (Figure 1D). These results indicate the ability of strain HUTR05 to generate positive effects on the initial stages of lettuce seedling growth, so the probiotic potential of this strain should be considered when improving the shoot length, as well as the number of leaves, the edible part of the plant which presents a greater economic interest. This is in accordance with the results of other authors who have observed these effects on other horticultural crop species inoculated with *Rhizobium* strains [19].

### 3.5. Effects on Lettuce Growth Promotion and Tolerance to Salinity Conditions

Various strains belonging to the genera *Bacillus*, *Pseudomonas*, or *Rhizobium* have been described to be capable of relieving and mitigating saline stress on crops, one of the most limiting abiotic stress conditions for the development of agricultural activity [11,20]. In addition to the efficient plant growth promotion, the inoculation of the crops with PGPR is a potential alternative.

Under greenhouse conditions, plants grown under normal conditions and inoculated with HUTR05 exhibited a more developed aerial part compared to the uninoculated plants, showing significantly 19% more leaves and a wet and dry weight of 91% and 21%, respectively, higher than the control treatment. Under salinity conditions, compared to the control treatments, the results were also favorable for the lettuces inoculated with HUTR05 exhibiting a number of leaves 4% higher and wet and dry weights 17% and 35% higher, respectively. In addition, the content of chlorophyll increased significantly up to 15% more than that in non-inoculated lettuces (Table 1).

Moreover, inoculation with HUTR05 under normal conditions improved the nutrient content of lettuce leaves compared to that in uninoculated plants (Table 2). Among the analyzed elements, the concentration of N and P was significantly higher in inoculated plants with respect to the control, being 7.2% and 24.6% higher, respectively. Additionally, under salinity conditions, Fe content was 24.7% higher in those plants inoculated with HUTR05, a statistically significant difference with respect to the control. Overall, these results indicate HUTR05’s potential to increase the nutritional content of lettuce plants, through the improvement in the efficiency of plant nutrient uptake. This fact may probably be the result of the effect generated by the PGPR mechanisms of this strain, tested under in vitro assays and whose genetic machinery has been previously described.

The obtained results coincide with the dynamics reported in the scientific literature on the ability of the *Rhizobium* genus to promote plant growth in non-leguminous plants [19], including *L. sativa* L. Although it is important to note that studies on this horticultural species were associated with other species of the bacterial genus, such as *R. leguminosarum* [22]. *R. laguerreae* has been shown to be capable of promoting plant development and food quality of certain horticultural crops, as well as tolerating high salt concentrations [10]. However, this is the first time that this species is reported to be capable of promoting lettuce growth and quality even under salinity stress conditions. This is a novelty of great interest for the development of new and potential bacterial biofertilizers since they help to tolerate these increasingly frequent adverse environmental conditions in agriculture, which undoubtedly deems an environmentally sustainable alternative to the pressing effects of global warming [15].

### 3.6. Analysis of Phenolic Compounds of Lactuca sativa L. Leaves

Table 3 and Table 4 show the content of phenolic acids and flavonoids, respectively, determined in lettuce leaves grown under the different conditions assayed. Phenolic acids account for ca. 73% of total phenolics compounds in lettuce leaves, whereas flavonoids represent ca. 27%, with flavonols being the main flavonoids detected (~25% of total phenolic compounds). This phenolic composition is in accordance with that previously reported for *Lactuca sativa* L. var romaine leaves [1]. Moreover, in this work, we have detected for the first time, although in minor levels (less than 1% of total phenolic composition), the presence of one apigenin derivative in lettuce leaves, which showed a [M-H]^−^ ion in mass spectrometry analysis at 449 whose main ion fragment in MS^2^ analysis was 269 [M-180-H]^−^.

The results of the two-way ANOVA (Table 5) indicated significant differences regarding the total content of both phenolic acids and flavonoids due to salinity, infection, and the interaction of both factors. This may point out, as explained below, that the effect of inoculation may be different depending on the level of saline stress of the media.

To be precise, it can be observed that saline stress is related to higher levels of phenolic compounds in lettuce leaves, since all the phenolic acids and flavonoids detected in this work, except for dicaffeoyl quinic acid, showed higher levels when lettuce is grown under saline conditions. Both, the content of total phenolic acids and that of flavonoid in lettuces grown under salinity conditions, showed a significant increase of ~130% when compared to lettuce grown under normal conditions. These results are in line with previous literature reporting that salinity can favor the biosynthesis of phenolic compounds in several food plants, including buckwheat, olive, or thyme [7,34,35]. However, studies about the effect of saline stress on the phenolic composition of lettuce have shown some conflicting results since there are diverse studies claiming increases in the phenolic composition and others that claim decreases in the levels of phenolic compounds because of saline stress. Some studies have reported that salinity can lead to non-significant changes if saline stress is low (NaCl concentration <100 mM ), or even to lower phenol index in lettuce leaves when high levels of salinity (150–1000 mM of NaCl) are used in growth media [5,36], thus, there could be a dose-dependent response. On the contrary, the results reported by Mahmoudi and co-workers [37] noted that lettuce can show different behaviors with regard to the accumulation of phenolic compounds depending not only on the lettuce variety but also on the type of salinity to which it is exposed [37]. These authors observed an increase of total phenolic content and total flavonoid content in romaine lettuces when they are grown for 12 days under 100 mM NaCl saline stress, which is in accordance with our results. In fact, among the consequences of the saline stress, an increase in the production of reactive oxygen species (ROS) in plants has been reported, which, in turn, implies an oxidative stress [6]. Hence, the increase in the phenolic compound levels in plants grown under saline conditions can be related to the activation of one of the ROS-scavenging mechanisms of plants, since phenolic compounds can play an important role in the protection against ROS generated under environmental stresses, because of their significant antioxidant activity [6]. Moreover, the presence of quercetin glycosides in quinoa and broad bean leaves have been related to (i) the scavenging of hydroxyl radicals formed in cytosol as response to salinity treatment, which can favor the retention of K+ and to (ii) the regulation of Na+ channels in plant cells, avoiding the accumulation of this ion in the cytosol, which in turn can prevent salt-stress-induced ROS formation [38]. Thus, the highest levels of flavonoids, mainly of quercetin 3-*O*-glucoside (increase under saline stress of ~180% compared to normal conditions) in lettuces grown under salinity conditions may be directly related to the response of lettuce to that stress.

As previously indicated, *R. laguerreae* inoculation also seems to affect the phenolic content of lettuce, since a significant increase was also observed in the total content of phenolic acids in leaves of lettuces inoculated with HUTR05 and grown under normal conditions. All the phenolic acids determined showed a significant increase in their levels with inoculation, with the caffeoyl acid derivatives (mainly dicaffeoyl quinic and cichoric acids) being the compounds showing the most important increases. However, when lettuces were grown under saline conditions, non-significant differences were found in the total content of phenolic acids when comparing inoculated and non-inoculated lettuces. Just some phenolic acids (caffeoyl malic, caffeoyl tartaric, and coumaric acids and procatechuic acid glucoside) showed significant increases whereas others, such as cichoric acid, showed a small although non-significant decrease in its content. Thus, as aforementioned, it seems that the effect of inoculation with HUTR05 is different depending on the salinity level of the media in which lettuce is grown. A similar trend was observed for flavonoid content in lettuce leaves. As in the case of phenolic acids, all flavonoids determined in lettuce leaves showed higher contents in lettuces inoculated with HUTR05 than in the non-inoculated ones when grown under normal conditions. On the contrary, when lettuce is grown under saline conditions, non-significant differences were found in the flavonoid content of the inoculated lettuces when compared to that of the non-inoculated ones. Hence, it seems that HUTR05 inoculation can promote the accumulation of phenolic compounds in lettuce leaves under normal growth conditions, but it seems not to have the same effect under saline conditions. PGPR had previously shown that it can promote the accumulation of both phenolic acids and flavonoids in other plants [39], even when grown under saline conditions [20]. Nevertheless, studies focused on lettuce showed that the effect of inoculation with different microorganisms is highly related to both, the type of inoculum and the lettuce variety. It has been reported that arbuscular mycorrhizal fungi inoculation in *L. sativa* L. var capitana does not have a significant effect on the phenolic content determined in lettuce leaves [2]. However, Avio and co-workers have studied the effect of two different arbuscular mycorrhizal fungi (*Funneliformis mosseae* and *Rhizoglomus irregulare*) on the phenolic content of two different cultivars of *L. sativa* L. var crispa, reporting that only *Rhizoglomus irregulare* promotes the accumulation of phenolic compounds in lettuce leaves compared to control samples [3]. These authors also reported significant phenolic content variety effects depending on the inoculum [3], highlighting again that the efficiency of inoculation can change according to the lettuce variety. Similarly, Santander and co-workers [16] have reported that the concentration of phenolic compounds in lettuce leaves was significantly affected not only by the cultivar and the inoculation with arbuscular mycorrhizal but also by the salinity level in growth conditions [16]. Both inoculums employed in this study lead to lower levels of phenolic compounds in lettuce when they were grown under saline conditions. They proposed that the microorganism can be useful for reducing the negative effect of salinity on the lettuce growth, which would be achieved by affecting the glycoside synthesis pathways, thus explaining the lower levels of phenolic compounds [16]. These results are in accordance with those reported in the present study so, it seems that HUTR05 inoculum can favor the growth of lettuce under saline conditions although the alleviation of saline stress may negatively affect the phenolic compound levels. However, under normal growth conditions the inoculation with *R. laguerreae* HUTR05 has proved to favor both the growth and the accumulation of phenolic compounds in lettuce leaves.

## 4. Conclusions

In the present work, the effects of the inoculation with a *Rhizobium laguerreae* strain in lettuce are analyzed for the first time. The ability of the HUTR05 strain to colonize the rhizosphere of *Lactuca sativa* L. and to increase the plant development and the nutritional content of this crop even under saline stress conditions, by improving the number of leaves, the shoot weight, and the content of certain bioactive compounds, in greenhouse conditions has been shown. The leaves of the lettuces inoculated with HUTR05 strain have shown significant increases in the content of phenolic acids, such as dicaffeoyl quinic and cichoric acids, as well as flavonoids, such as quercetin 3-*O*-glucoside. Moreover, a relevant enhancement in nitrogen or phosphorus content has been also observed. Thus, these results show the potential of *R. laguerreae* HUTR05 to be added in non-legume crops, due to its ability to promote plant development, to relieve saline stress effects, and to improve plant nutritional content and its health potential. The use of bacterial biofertilizers deems an advantage over traditional farming techniques based on the application of chemical fertilizers, as well as an environmentally sustainable alternative related to the new trends in healthy consumption.

## Figures and Tables

**Table 1:** Results from in vitro growth promotion assays and from greenhouse growth promotion assays and chlorophyll content, in lettuce inoculated with *Rhizobium laguerreae* HUTR05 strain.

**In Vitro Growth Promotion Assays**								
**Treatment**	**Shoot Length** **(±S.E.) (mm)**	**Root Length** **(±S.E.) (mm)**	**Number of Secondary Roots** **(±S.E.)**	**Number of Leaves** **(±S.E.)**	**Shoot Length** **(±S.E.) (mm)**	**Root Length** **(±S.E.) (mm)**	**Number of Secondary Roots (±S.E.)**	**Number of Leaves** **(±S.E.)**
	**7 dpi**				**14 dpi**			
**Control**	19.91 (±0.85)	24.61 (±0.83)	2.75 (±0.17)	2.73 (±0.12)	26.94 (±0.88)	59.68 (±2.64)	3.32 (±0.21)	3.67 (±0.11)
**HUTR05**	21.25 (±0.48)	28.77 (±1.58) *	2.62 (±0.21)	3.00 (±0.00) *	26.68 (±1.25)	56.50 (±3.02)	4.13 (±0.36) *	3.90 (±0.07)
**Greenhouse Assays**								
**Treatment**	**Chlorophyll** **(±S.E.) (SPAD Units)**	**Number of leaves** **(±S.E.)**	**SFW** **(±S.E.) (g)**	**SDW** **(±S.E.) (g)**	**Chlorophyll** **(±S.E.) (SPAD Units)**	**Number of leaves** **(±S.E.)**	**SFW** **(±S.E.) (g)**	**SDW** **(±S.E.) (g)**
	**Non-saline conditions**				**100 mM NaCl**			
**Control**	25.34 (±0.72)	5.53 (±0.23)	2.39 (±0.26)	0.14 (±0.01)	25.54 (±0.54)	5.29 (±0.17)	2.95 (±0.18)	0.12 (±0.01)
**HUTR05**	26.00 (±0.53)	6.56 (±0.25) *	4.54 (±0.28) *	0.17 (±0.01)	29.32 (±0.69) *	5.53 (±0.19)	3.46 (±0.16)	0.16 (±0.01) *

Values followed by * are significantly different from its respective control according to Fisher’s protected LSD (least significant differences) (*p* ≤ 0.05). S.E. = standard error; dpi = days post inoculation; SFW = shoot fresh weight; SDW = shoot dry weight.

**Table 2:** Effects of *Rhizobium laguerreae* HUTR05 inoculation on nutrient contents of lettuce plants.

	Normal Conditions		100 mM NaCl	
	Control (±S.E.)	HUTR05 (±S.E.)	Control (±S.E.)	HUTR05 (±S.E.)
N (%)	5.68 (±0.15)	6.09 (±0.06) *	5.31 (±0.05) #	5.22 (±0.02)
Ca (g/plant kg)	13.38 (±1.04)	14.28 (±0.25)	11.30 (±0.21) #	10.48 (±0.56)
Fe (mg/plant kg)	225.39 (±139.42)	132.80 (±4.17)	109.89 (±15.94)	137.07 (±16.53)
K (g/plant kg)	92.69 (±1.19)	110.85 (±4.15)	99.08 (±7.34)	91.89 (±3.64)
Mg (g/plant kg)	6.21 (±0.89)	7.34 (±0.29)	5.21 (±0.09)	4.89 (±0.29)
Mn (mg/plant kg)	46.36 (±9.46)	49.45 (±2.19)	47.217 (±1.91)	45.88 (±2.18)
Na (g/plant kg)	3.23 (±0.32)	3.58 (±0.11)	22.75 (±0.41) #	22.68 (±0.83)
P (g/plant kg)	8.10 (±0.27)	10.09 (±0.12) *	8.75 (±0.24)	7.95 (±0.22)

* within the same row and within each type of conditions (non-saline and saline conditions) indicates significant differences from its respective control, # within the 100 mM NaCl control column indicates significant differences from normal conditions control, both according to Fisher’s protected LSD (least significant differences) (*p* ≤ 0.05). S.E. = standard error.

**Table 3:** Phenolic acid content (g kg^−1^) of control lettuce plants and plants inoculated with *Rhizobium laguerreae* HUTR05.

Phenolics Acids (g kg^−1^)	Normal Conditions		100 mM NaCl	
	Control (±S.E.)	HUTR05 (±S.E.)	Control (±S.E.)	HUTR05 (±S.E.)
Caffeoyl malic acid	1.690 (±0.032)	1.914 (±0.013) *	1.741 (±0.087)	2.013 (±0.002) *
Caffeoyl quinic acids	3.566 (±0.018)	5.080 (±0.023) *	4.490 (±0.245) #	4.691 (±0.018)
Caffeoyl tartaric acid	1.868 (±0.013)	2.417 (±0.010) *	3.697 (±0.163) #	4.172 (±0.043) *
Cichoric acid (dicaffeoyltartaric acid)	10.293 (±0.056)	16.893 (±0.177) *	12.682 (±0.734) #	12.452 (±0.109)
Coumaric acid	0.390 (±0.009)	0.520 (±0.013) *	0.506 (±0.035) #	0.588 (±0.007) *
Dicaffeoyl quinic acid	0.765 (±0.016)	1.610 (±0.014) *	0.724 (±0.038)	0.797 (±0.015)
Protocatechuic acid glucoside	0.370 (±0.012)	0.664 (±0.021) *	0.522 (±0.054) #	0.830 (±0.010) *
Total phenolics acids	18.942 (±0.079)	29.097 (±0.134) *	24.361 (±1.352) #	25.542 (±0.156)

* within the same row and within each type of conditions (non-saline and saline conditions) indicates significant differences from its respective control, # within the 100 mM NaCl control column indicates significant differences from normal conditions control, both according to Fisher’s protected LSD (least significant differences) (*p* ≤ 0.05). S.E. = standard error.

**Table 4:** Flavonoid content (g kg^−1^) of control lettuce plants and plants inoculated with *Rhizobium laguerreae* HUTR05.

Flavonoids (g kg^−1^)	Normal Conditions		100 mM NaCl	
	Control (±S.E.)	HUTR05 (±S.E.)	Control (±S.E.)	HUTR05 (±S.E.)
Apigenin derivative	0.137 (±0.001)	0.449 (±0.018) *	0.229 (±0.014) #	0.717 (±0.009) *
Luteolin 7-*O*-glucoside	0.231 (±0.003)	0.341 (±0.010) *	0.324 (±0.021) #	0.314 (±0.006)
Quercetin 3-*O*-glucuronide	2.370 (±0.014)	3.758 (±0.056) *	2.862 (±0.145) #	2.436 (±0.007)
Quercetin 3-*O*-malonyl glucoside	3.185 (±0.025)	4.597 (±0.055) *	4.115 (±0.093) #	3.745 (±0.021)
Quercetin 3-*O*-glucoside	0.810 (±0.010)	2.732 (±0.042) *	1.437 (±0.082) #	1.417 (±0.001)
Total flavonoids	6.733 (±0.046)	11.877 (±0.076) *	8.967 (±0.339) #	8.629 (±0.039)

* within the same row and within each type of conditions (non-saline and saline conditions) indicates significant differences from its respective control, # within the 100 mM NaCl control column indicates significant differences from normal conditions control, both according to Fisher’s protected LSD (least significant differences) (*p* ≤ 0.05 ). S.E. = standard error.

**Table 5:** Results of two-way ANOVA performed to assess the effect of salinity and inoculation on the total content of phenolic acids and flavonoids in lettuce leaves.

	Total Phenolic Content		Total Flavonoid Content	
	F-Value	*p*-Value	F-Value	*p*-Value
Salinity	5.555	0.0462	557.3	0.0011
Inoculation	205.5	<0.0001	24.81	<0.0001
Interaction salinity × inoculation	128.8	<0.0001	725.1	<0.0001

## References

[1] Llorach R., Martínez-Sánchez A., Tomás-Barberán F.A., Gil M.I., Ferreres F. (2008). Characterisation of polyphenols and antioxidant properties of five lettuce varieties and escarole. Food Chem..

[2] Baslam M., Garmendia I., Goicoechea N. (2013). The arbuscular mycorrhizal symbiosis can overcome reductions inyield and nutritional quality in greenhouse-lettuces cultivated atinappropriate growing seasons. Sci. Hortic..

[3] Avio L., Sbrana C., Giovannetti M., Frassinetti S. (2017). Arbuscular mycorrhizal fungi affect total phenolics content and antioxidant activity in leaves of oak leaf lettuce varieties. Sci. Hortic..

[4] Shatilov M.V., Razin A.F., Ivanova M.I. (2019). Analysis of the world lettuce market. IOP Conf. Ser. Earth Environ. Sci..

[5] Ahmed S., Ahmed S., Roy S.K., Woo S.H., Sonawane K.D., Shohael A.M. (2019). Effect of salinity on the morphological, physiological and biochemical properties of lettuce (*Lactuca sativa* L.) in Bangladesh. Open Agric..

[6] Waśkiewicz A., Muzolf-Panek M., Goliński P., Ahmad P., Azooz M., Prasad M. (2013). Phenolic content changes in plants under salt stress. Ecophysiology and Responses of Plants under Salt Stress.

[7] Lim J.H., Park K.J., Kim B.K., Jeong J.W., Kim H.J. (2012). Effect of salinity stress on phenolic compounds and carotenoids in buckwheat (*Fagopyrum esculentum* M.) sprout. Food Chem..

[8] Dueñas M., Martínez-Villaluenga C., Limón R.I., Peñas E., Frias J. (2015). Effect of germination and elicitation on phenolic composition and bioactivity of kidney beans. Food Res. Int..

[9] Kaushal M., Wani S.P. (2016). Rhizobacterial-plant interactions: Strategies ensuring plant growth promotion under drought and salinity stress. Agric. Ecosyst. Environ..

[10] Benidire L., Lahrouni M., Daoui K., Fatemi Z.A., Carmona R.G., Goöttfert M., Oufdou K. (2017). Phenotypic and genetic diversity of Moroccan rhizobia isolated from *Vicia faba* and study of genes that are likely to be involved in their osmotolerance. Syst. Appl. Microbiol..

[11] Bano A., Fatima M. (2009). Salt tolerance in *Zea mays* (L.) following inoculation with *Rhizobium* and *Pseudomonas*. Biol. Fertil. Soil.

[12] Roubtsova T.V., Bostock R.M. (2009). Episodic abiotic stress as a potential contributing factor to onset and severity of disease caused by *Phytophthora ramorum* in *Rhododendron* and *Viburnum*. Plant Dis..

[13] Egamberdieva D., Lugtenberg B., Miransari M. (2014). Use of plant growth-promoting rhizobacteria to alleviate salinity stress in plants. Use of Microbes for the Alleviation of Soil Stresses.

[14] Radhakrishnan R., Baek K.H. (2017). Physiological and biochemical perspectives of non-salt tolerant plants during bacterial interaction against soil salinity. Plant Physiol. Biochem..

[15] Niu G., Davis T.D., Masabni J. (2019). A review of salinity tolerance research in horticultural crops. J. Arid Land Stud..

[16] Santander C., Ruiz A., García S., Aroca R., Cumming J., Cornejo P. (2020). Efficiency of two arbuscular mycorrhizal fungal inocula to improve saline stress tolerance in lettuce plants by changes of antioxidant defense mechanisms. J. Sci. Food Agric..

[17] Flores-Félix J.D., Velázquez E., García-Fraile P., González-Andrés F., Silva L.R., Rivas R. (2018). *Rhizobium* and *Phyllobacterium* bacterial inoculants increase bioactive compounds and quality of strawberries cultivated in field conditions. Food Res. Int..

[18] Jiménez-Gómez A., Flores-Félix J.D., García-Fraile P., Mateos P.F., Menéndez E., Velázquez E., Rivas R. (2018). Probiotic activities of *Rhizobium laguerreae* on growth and quality of spinach. Sci. Rep..

[19] Maurya R., Verma S., Bahadur I., Kumar A., Meena V.S. (2019). Advances in the application of plant growth-promoting rhizobacteria in horticulture. Plant Growth Promoting Rhizobacteria for Agricultural Sustainability.

[20] Jiménez-Gómez A., García-Estévez I., García-Fraile P., Escribano-Bailón M.T., Rivas R. (2020). Increase in phenolics compounds of *Coriandrum sativum* L. after the application of a *Bacillus halotolerans* biofertilizer. J. Sci. Food Agric..

[21] Seshadri R., Reeve W.G., Ardley J.K., Tennessen K., Woyke T., Kyrpides N.C., Ivanova N.N. (2015). Discovery of novel plant interaction determinants from the genomes of 163 root nodule bacteria. Sci. Rep..

[22] García-Fraile P., Carro L., Robledo M., Ramírez-Bahena M.H., Flores-Félix J.D., Fernández M.T., Mateos P.F., Rivas R., Igual J.M., Martínez-Molina E. (2012). *Rhizobium* promotes non-legumes growth and quality in several production steps: Towards a biofertilization of edible raw vegetables healthy for humans. PLoS ONE.

[23] Rivas R., García-Fraile P., Mateos P.F., Martínez-Molina E., Velázquez E. (2007). Characterization of xylanolytic bacteria present in the bract phyllosphere of the date palm *Phoenix dactylifera*. Lett. Appl. Microbiol..

[24] Flores-Félix J.D., Sánchez-Juanes F., García-Fraile P., Valverde A., Mateos P.F., Gónzalez-Buitrago J.M., Velázquez E., Rivas R. (2019). *Phaseolus vulgaris* is nodulated by the symbiovar *viciae* of several genospecies of *Rhizobium laguerreae* complex in a Spanish region where *Lens culinaris* is the traditionally cultivated legume. Syst. Appl. Microbiol..

[25] Zerbino D.R., Birney E. (2008). Velvet: Algorithms for de novo short read assembly using de Bruijn graphs. Genome Res..

[26] Overbeek R., Olson R., Pusch G.D., Olsen G.J., Davis J.J., Disz T., Edwars R.A., Gerdes S., Parrello B., Shukla M. (2014). The SEED and the rapid annotation of microbial genomes using subsystems technology (RAST). Nucleic Acids Res..

[27] Pikovskaya R.I. (1948). Mobilization of phosphorus in soil in connection with vital activity of some microbial species. Mikrobiologiya.

[28] Nautiyal C.S. (1999). An efficient microbiological growth medium for screening phosphate solubilizing microorganisms. FEMS Microbiol. Lett..

[29] Alexander D.B., Zuberer D.A. (1991). Use of chrome azurol S reagents to evaluate siderophore production by rhizosphere bacteria. Biol. Fertil. Soils.

[30] Flores-Félix J.D., Menéndez E., Rivera L.P., Marcos-García M., Martínez-Hidalgo P., Mateos P.F., Martínez-Molina E., Velázquez M.D.L.E., García-Fraile P., Rivas R. (2013). Use of *Rhizobium leguminosarum* as a potential biofertilizer for *Lactuca sativa* and *Daucus carota* crops. J. Plant Nutr. Soil Sci..

[31] Saïdi S., Ramírez-Bahena M.H., Santillana N., Zuniga D., Álvarez-Martínez E., Peix A., Mhamdi R., Velázquez E. (2014). *Rhizobium laguerreae* sp. nov. nodulates *Vicia* faba on several continents. Int. J. Syst. Evolut. Microbiol..

[32] Wang D., Couderc F., Tian C.F., Gu W., Liu L.X., Poinsot V. (2018). Conserved composition of *nod* factors and exopolysaccharides produced by different phylogenetic lineage *Sinorhizobium* strains nodulating soybean. Front. Microbiol..

[33] Menéndez E., Robledo M., Jiménez-Zurdo J.I., Velázquez E., Rivas R., Murray J.D., Mateos P.F. (2019). Legumes display common and host-specific responses to the rhizobial cellulase CelC2 during primary symbiotic infection. Sci. Rep..

[34] Petridis A., Therios I., Samouris G., Tananaki C. (2012). Salinity-induced changes in phenolic compounds in leaves and roots of four olive cultivars (*Olea europaea* L.) and their relationship to antioxidant activity. Environ. Exp. Bot..

[35] Bistgani Z.E., Hashemi M., DaCosta M., Craker L., Maggi F., Morshedloo M.R. (2019). Effect of salinity stress on the physiological characteristics, phenolic compounds and antioxidant activity of *Thymus vulgaris* L. and *Thymus daenensis* Celak. Ind. Crops Prod..

[36] Kim H.J., Fonseca J.M., Choi J.H., Kubota C., Kwon D.Y. (2008). Salt in irrigation water affects the nutritional and visual properties of romaine lettuce (*Lactuca sativa* L.). J. Agric. Food Chem..

[37] Mahmoudi H., Huang J., Gruber M.Y., Kaddour R., Lachaâl M., Ouerghi Z., Hannoufa A. (2010). The impact of genotype and salinity on physiological function, secondary metabolite accumulation, and antioxidative responses in lettuce. J. Agric. Food Chem..

[38] Ismail H., Maksimovic J.D., Maksimovic V., Shabala L., Živanovic B.D., Tian Y., Jacobsen S.E., Shabala S. (2016). Rutin, a flavonoid with antioxidant activity, improves plant salinity tolerance by regulating K^+^ retention and Na^+^ exclusion from leaf mesophyll in quinoa and broad beans. Funct. Plant Biol..

[39] Nautiyal C.S., Govindarajan R., Lavania M., Pushpangadan P. (2008). Novel mechanism of modulating natural antioxidants in functional foods: Involvement of plant growth promoting rhizobacteria NRRL B-30488. J. Agric. Food Chem..

